# Torsade de pointes induced by intravenous amiodarone therapy accompanied by marked augmentation of the transmural dispersion of repolarization in a patient with tachycardia‐induced‐cardiomyopathy

**DOI:** 10.1111/anec.12810

**Published:** 2020-10-18

**Authors:** Ryo Yonai, Mihoko Kawabata, Shingo Maeda, Tomoyuki Kawashima, Yasuhide Tsuda, Takashi Nakasone, Hiroki Nakane, Kenzo Hirao

**Affiliations:** ^1^ Division of Cardiovascular Medicine AOI Universal Hospital Kanagawa Japan

**Keywords:** amiodarone, hemodialysis, tachycardia‐induced‐cardiomyopathy, torsade de pointes, Tpeak‐Tend interval, transmural dispersion of repolarization

## Abstract

We report a 77‐year‐old human on renal dialysis for end‐stage renal disease with heart failure and atrial fibrillation (AF) complicated by a high ventricular frequency. The underlying disease was thought as tachycardia‐induced‐cardiomyopathy. Intravenous infusion of amiodarone was initiated, and direct current cardioversion succeeded in converting AF to sinus rhythm. Then, excessive increases in the QT and Tpeak‐Tend (Tp‐e) intervals were seen and hypokalemia induced by hemodialysis led to the development of numerous episodes of torsades de pointes (TdP). Magnesium repletion was effective in preventing TdP, while Tp‐e intervals returned to the previous values 2 days after the discontinuation of amiodarone.

## 1 CASE REPORT

**FIGURE 1:**
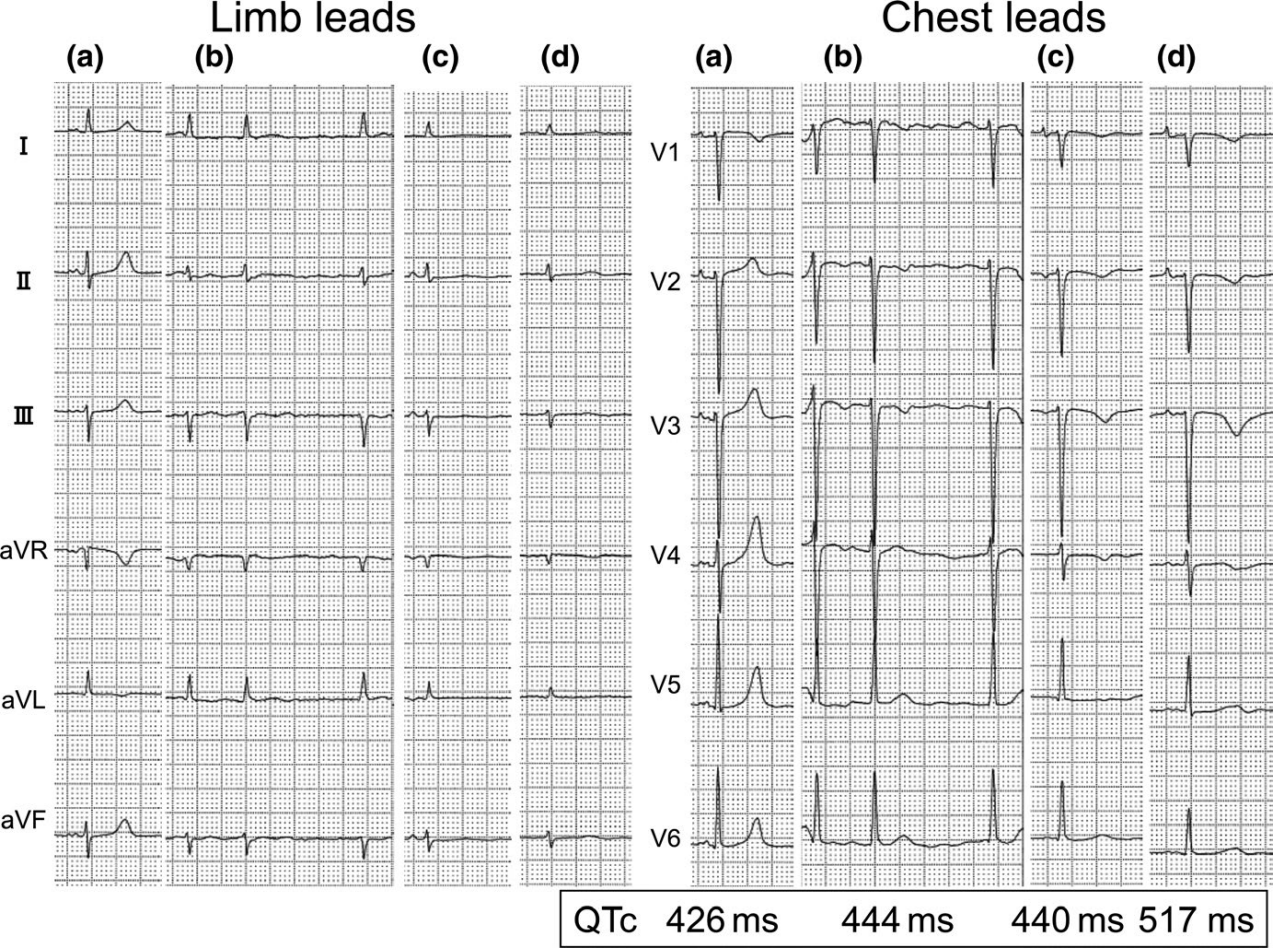
The serial ECGs from 6 months prior to admission (a), on admission (b), just after successful direct current cardioversion with 4 hr of administration of intravenous amiodarone (c), and 15 hr after beginning amiodarone (d). (a) The rhythm was sinus rhythm (SR) with QTc interval of 426 ms. T waves were positive and symmetrical, and poor R wave progression was seen. (b) The rhythm was atrial fibrillation with a heart rate in the 100 s and QTc interval of 444 ms. Negative T waves in precordial leads newly developed. (c) Just after the recovery to SR, QTc interval was 440 ms. Negative T waves in the precordial leads were symmetrical with Tpeak‐Tend (Tp‐e) interval of 40 ms and corrected‐Tp‐e (c‐Tp‐e) interval of 42 ms. (d) QTc interval prolonged to 517 ms, which was accompanied by prolonged Tp‐e interval of 240 ms (c‐Tp‐e interval: 255 ms)

A 77‐year‐old human on regular hemodialysis for end‐stage renal disease was admitted because of heart failure. He had been well until persistent atrial fibrillation (AF) with rapid ventricular response started 2 months prior. His past medical history included gastric cancer and bile duct cancer surgeries. ECG on admission revealed AF with a heart rate in the 100 s, and poor R wave progression with newly developed negative T waves in the precordial leads (Figure 1b), however, coronary angiography revealed no stenosis. Chest radiography confirmed left‐sided pleural effusion. Transthoracic echocardiography revealed diffuse left ventricular (LV) hypokinesis, with ejection fraction of 25%, which was 58% 3 months prior during sinus rhythm (SR). As the rapid AF was sustained and there were no other causes of the LV dysfunction, tachycardia‐induced‐LV dysfunction and heart failure were suspected. Transesophageal echocardiography revealed no intracardiac thrombus; then, intravenous amiodarone was initiated, and DC cardioversion succeeded in converting the AF to SR (Figure 1c). The QT intervals were measured manually with calipers in all 12 leads. They were defined as the time interval between the earliest deflection of the QRS complex and the point of T‐wave offset, which was defined by the return of the terminal T wave to the isoelectric baseline. When U waves were present, U waves were excluded using the presented guidelines (Lepechkin & Surawicz, 1952), therefore, QT interval was measured to the nadir of the curve between the T wave and U wave. Biphasic T waves were distinguished from U waves by comparison with similar complexes in contiguous ECG leads. If the end of the T wave could not be reliably determined or when the T waves were isoelectric or of low amplitude, QT measurements were not made and these leads were excluded from analysis. Then, T‐wave peak was determined, and the Tpeak‐Tend (Tp‐e) intervals from T‐wave peak to T‐wave end were measured. In the case of negative or biphasic T waves, T‐wave peak was defined to the nadir of the T wave. T waves smaller than 1.5 mm in amplitude were not measured. The Tp‐e value reported was the maximum. All measured QT and Tp‐e intervals were corrected (QTc and c‐Tp‐e) for heart rate using the Bazett formula (QTc; QT/√RR, c‐Tp‐e; Tp‐e/√RR). QTc interval was 444 ms during AF on admission, and after the conversion to SR, QTc interval was 440 ms and c‐Tp‐e 42 ms.

**FIGURE 2:**
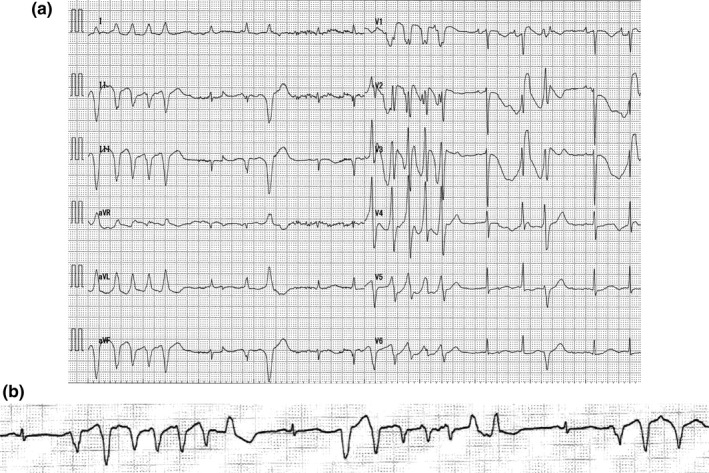
12‐lead ECG (a) and ECG monitor strips (b) after hemodialysis. After regular hemodialysis, mild hypokalemia was induced. (a) Note the remarkably bizarre negative T waves in the precordial leads, which did not end when the next sinus rhythm QRS complex started. Therefore, QT or Tp‐e intervals could not be measured. In the 5th beat, torsade de pointes (TdP) terminated spontaneously. (b) The patient developed repetitive short‐lasting TdP episodes terminating spontaneously. A “short‐long‐short” sequence preceded the onset of TdP

**FIGURE 3:**
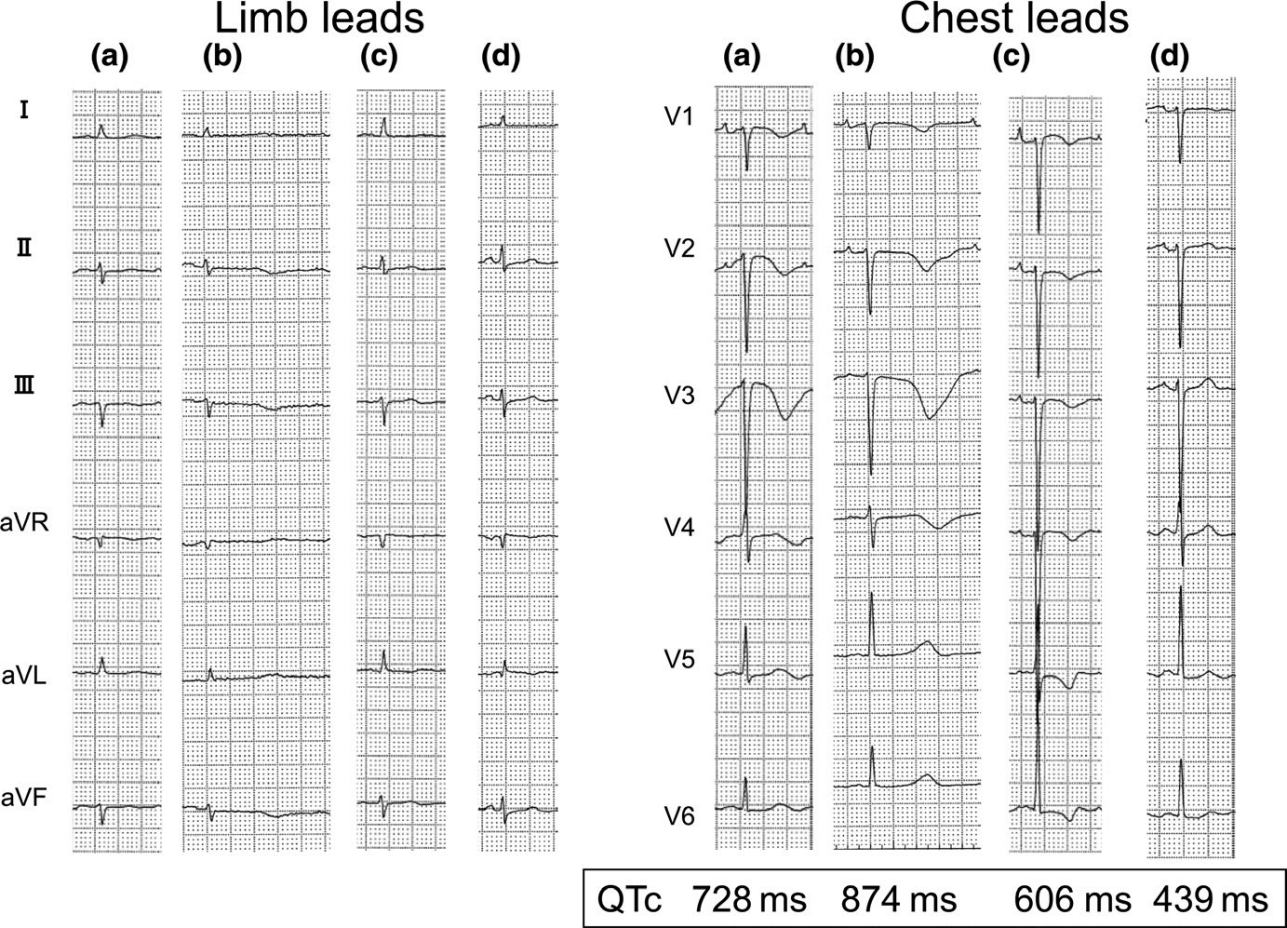
The serial ECGs from 10 hr after the discontinuation of amiodarone (a), 1 day after the discontinuation (b), 2 days after the discontinuation (c), and 1 month later (d). (a, b) T waves in the precordial leads were still negative and corrected‐ Tpeak‐Tend (c‐Tp‐e interval) was prolonged (291 and 341 ms, each). (c) Negative T waves were seen in all chest leads; however, they were symmetrical. (d) T waves in all chest leads were positive and symmetrical

ECG taken 15 hr after beginning amiodarone revealed that QTc interval prolonged of 517 ms, which was accompanied by prolonged c‐Tp‐e interval of 255 ms (Figure 1d), with normal serum electrolytes. Therefore, the intravenous amiodarone was ceased (total dose of 370 mg). However, QTc and c‐Tp‐e interval did not recover; on the contrary, both prolonged even further after hemodialysis, which triggered hypokalemia (3.3 mEq/L). The patient developed repetitive short‐lasting torsade de pointes tachycardias (TdPs) terminating spontaneously (Figure 2). During the consecutive TdP episodes, he had syncope once. Magnesium repletion was effective in preventing TdP; however, T waves became symmetrical and c‐Tp‐e intervals shortened 2 days after the discontinuation of amiodarone (Figure 3). During the TdP episodes, isoproterenol could not be administered for fear of inducing AF. Following catheter ablation of AF, he kept SR. No TdP recurred, and the patient remained asymptomatic. Three months later, catheter ablation of atrial tachycardia which occurred newly was performed. LV function recovered 8 months later.

## 2 DISCUSSION

Amiodarone is widely used for the treatment of malignant arrhythmias with a remarkably low frequency of proarrhythmia. The incidence of TdP associated with oral amiodarone is reported to be <1.0% (Hohnloser et al., 1994), while that with intravenous amiodarone is about 1.5% (Shenthar et al., 2017). Although both amiodarone and other antiarrhythmic drugs including class Ia and other class III prolong the QT interval, TdP is overwhelmingly rare during amiodarone therapy. The mechanism of the difference is that amiodarone homogeneously prolongs the ventricular repolarization, whereas other antiarrhythmic drugs prolong it in a nonhomogeneous fashion accompanied by an increase in QT interval dispersion (Friedman & Stevenson, 1998; Hii et al., 1992; Milberg et al., 2004; van Opstal et al., 2001). Antezelvich introduced the concept of Tp‐e interval in surface ECG as an index of transmural dispersion of repolarization (TDR) based on the studies with the coronary‐perfused wedge preparation, that repolarization of the epicardial action potential coincides with the peak of the T wave and repolarization of the mid‐myocardial cells (M cells) is coincident with the end of the T wave, so that Tp‐e interval provides a measure of TDR, with forecasting risk for the development of TdP (Sicouri & Antzelevitch, 1991). Studies have indicated that drugs that do not increase TDR have little or no potential to induce TdP despite causing a prolongation of the QT interval. Amiodarone has in common the ability to block IKs, IKr, and late INa. This combination produces a preferential prolongation of APD of the epicardium and endocardium so that the QT interval is prolonged, but the TDR is actually reduced and TdP rarely, if ever, occurs under these conditions (Hii et al., 1992; Kotake et al., 2015; Milberg et al., 2004; van Opstal et al., 2001).

The case reports of amiodarone‐induced‐TdP so far were rare and have shown significant QT prolongation, but without Tp‐e prolongation (Belardinelli et al., 2003; Friedman & Stevenson, 1998; Hii et al., 1992; Hohnloser et al., 1994; Kotake et al., 2015; Shenthar et al., 2017). The increased TDR could provide a more accurate electrophysiologic marker of the risk for TdP than does the QT interval. In this case with tachycardia‐induced‐cardiomyopathy (TCM) due to rapid AF, the QT interval prolonged dramatically with bizarre T waves suggesting augmented TDR after the administration of intravenous amiodarone. Finally, hypokalemia caused by hemodialysis initiated TdP accompanied by syncope. To the best of our knowledge, this is the first report of TdP with marked Tp‐e prolongation in a patient during amiodarone therapy.

Although the mechanism responsible for the marked augmentation of TDR with amiodarone in our case is unclear, it would be speculated as multifactorial. TDR is increased by drugs, heart failure, acute myocardial infarction, and various channelopathies. First, we previously reported the possibility that the transmural heterogeneity of myocardial ischemia might influence the repolarization resulting in increased TDR (Kawabata et al., 2008). Although CAG revealed no lesions in this case, the possibility of myocardial ischemia in microvascular level could not be completely ruled out. Second, as both QT and Tp‐e intervals were not prolonged before the administration of amiodarone in this case, the marked QT/TDR increase was induced by amiodarone; however, the possibility of acquired long QT syndrome based on the genetic causes could not be excluded as a genetic test was not performed. Third, the underlying disease was thought as TCM in this patient. It is reported that in tachycardia‐induced heart failure K+ currents are down‐regulated ununiformly, causing enhanced TDR (Akar et al., 2005). It was speculated that the underlying molecular features in TCM would influence the augmentation of TDR in this case. Forth, a different distribution or clearance of amiodarone could be related. In previous report of animal models of acquired long QT syndrome, amiodarone increased QTc time in 6 of 7 dogs, while dispersion of repolarization was increased in 3. The three dogs tended to have higher tissue levels of amiodarone and its metabolite compared with those without dispersion of repolarization (van Opstal et al., 2001). In this case, we did not check the concentration of amiodarone or n‐desethylamiodarone; however, there was a possibility that they were quite high.

## 3 CONCLUSION

Although the reported incidence of TdP during amiodarone therapy is low, careful ECG monitoring should be undergone to check not only QT interval but Tp‐e interval.

## CONFLICT OF INTEREST

None.

## ETHICAL APPROVAL

The authors have obtained the patient's informed consent.

## Data Availability

The data that support the findings of this study are available from the corresponding author upon reasonable request.
